# Comprehensive Physio-Biochemical Evaluation Reveals Promising Genotypes and Mechanisms for Cadmium Tolerance in Tibetan Hull-Less Barley

**DOI:** 10.3390/plants13243593

**Published:** 2024-12-23

**Authors:** Md Rafat Al Foysal, Cheng-Wei Qiu, Jakkrit Sreesaeng, Saad Elhabashy, Delara Akhter, Shuo Zhang, Shou-Heng Shi, Feibo Wu

**Affiliations:** 1 Department of Agronomy, College of Agriculture and Biotechnology, Zijingang Campus, Zhejiang University, Hangzhou 310058, China; rafat.aha@sau.ac.bd (M.R.A.F.); jakkrit@zju.edu.cn (J.S.); saad.elhabshy@alexu.edu.eg (S.E.); zmike@zju.edu.cn (S.Z.); shouhengshi@zju.edu.cn (S.-H.S.); wufeibo@zju.edu.cn (F.W.); 2 Department of Agronomy and Haor Agriculture, Faculty of Agriculture, Sylhet Agricultural University, Sylhet 3100, Bangladesh; 3 Expert Centre of Innovative Agriculture, Thailand Institute of Scientific and Technological Research, Pathum Thani 12120, Thailand; 4 Department of Crop Science, Faculty of Agriculture, Alexandria University, Alexandria 21545, Egypt; 5 Department of Genetics and Plant Breeding, Sylhet Agricultural University, Sylhet 3100, Bangladesh; delara.gpb@sau.ac.bd

**Keywords:** hull-less barley, cadmium tolerance, integrated score, photosynthesis, oxidative stress, antioxidant enzyme

## Abstract

Cadmium (Cd) toxicity in agricultural soil is increasing globally and significantly impacts crop production and food safety. Tibetan hull-less barley (*Hordeum vulgare* L. var. *nudum*), an important staple food and economic crop, exhibits high genetic diversity and is uniquely adapted to the harsh conditions of the Qinghai–Tibet Plateau. This study utilized hydroponic experiments to evaluate the genotypic differences in Cd tolerance among 71 Tibetan hull-less barley genotypes. Physiological assessments revealed significant reductions in various growth parameters under Cd stress compared to normal conditions: soil–plant analysis development (SPAD) value, shoot height, root length, shoot and root fresh weight, shoot and root dry weight, of 11.74%, 39.69%, 48.09%, 52.88%, 58.39%, 40.59%, and 40.52%, respectively. Principal component analysis (PCA) revealed key traits contributing to Cd stress responses, explaining 76.81% and 46.56% of the variance in the preliminary and secondary selection. The genotypes exhibited varying degrees of Cd tolerance, with X178, X192, X215, X140, and X162 showing high tolerance, while X38 was the most sensitive based on the integrated score and PCA results. Validation experiments confirmed X178 as the most tolerant genotype and X38 as the most sensitive, with observed variations in morphological, physiological, and biochemical parameters, as well as mineral nutrient responses to Cd stress. Cd-tolerant genotypes exhibited higher chlorophyll content, net photosynthesis rates, and effective photochemical capacity of photosystem II, along with an increased Cd translocation rate and reduced oxidative stress. This was accompanied by elevated activities of antioxidant enzymes, including superoxide dismutase (SOD), peroxidase (POD), and catalase (CAT), indicating a robust stress response mechanism. These findings could facilitate the development of high-tolerance cultivars, with X178 as a promising candidate for further research and cultivation in Cd-contaminated soils.

## 1. Introduction

Cadmium (Cd) toxicity has emerged as a critical environmental issue globally, significantly threatening agricultural production and food safety [1]. The rise in soil Cd pollution is primarily due to industrial activities, such as steel manufacturing, sewage irrigation, the use of Cd-containing phosphate fertilizers and pesticides, and municipal waste [2,3,4]. Elevated Cd levels in plants can lead to metabolic disruptions, growth inhibition, and reduced biomass production by interfering with critical physiological processes, including photosynthesis, enzyme activity, reactive oxygen species (ROS) management, and nutrient uptake [5,6]. The accumulation of Cd in edible plant tissues also poses a serious risk to human health through the food chain, potentially causing chronic health conditions such as kidney tubule damage, rhinitis, and emphysema [7,8]. A well-documented case of chronic Cd poisoning is the outbreak of “Itai-Itai disease” in Japan in the last century. Plants have evolved several adaptive mechanisms to mitigate Cd stress, such as restricting Cd transport to shoot tissues, detoxifying and sequestering Cd in vacuoles, activating antioxidant defenses, and modulating hormone levels [9,10]. Recent surveys indicate that 7% of agricultural soils in China are contaminated with Cd [11]. Therefore, understanding how plants respond to Cd stress and the mechanisms of Cd translocation is crucial for developing Cd-tolerant crop varieties with low-grain Cd accumulation, a key strategy for improving agricultural yields and ensuring food safety [12,13,14].

Barley (*Hordeum vulgare* L.), one of the first domesticated cereals and the fourth most widely cultivated crop globally, is used for human food, animal feed, and malting, making it a significant source of Cd in the human diet [15,16,17]. However, the threat of heavy metal contamination, particularly Cd, poses serious risks to barley cultivation. To address this challenge, the selection of Cd-tolerant barley genotypes is crucial for maintaining agricultural sustainability and securing food systems from the detrimental effects of Cd toxicity. Barley is also a valuable model plant in research on heavy metal pollution [18]. Recent studies have extensively explored the genetic diversity within barley populations to identify genotypes with inherent Cd tolerance, offering promising prospects for sustainable agriculture in polluted environments. Several genes have been implicated in Cd tolerance and accumulation in barley. For example, HvNramp5 (Natural Resistance-Associated Macrophage Protein 5) is a key transporter regulating Cd uptake in roots [19]. HvHMA3 (Heavy Metal ATPase 3), located on the vacuolar membrane of root cells, functions to translocate Cd from the cytoplasm to the vacuole, thereby acting as a Cd compartmentalizer [20]. Our previous research on Cd-tolerant and Cd-sensitive genotypes has successfully identified and functionally characterized several novel genes associated with Cd tolerance and accumulation, including *HvPAA1* (encoding P-Type ATPase 1) [11], *HvNAT2* (encoding Nucleobase-Ascorbate Transporter 2) [3], and *HvGAMYB* (encoding a gibberellin-responsive MYB transcription factor) [21]. Hull-less barley, an ancient cereal and a crucial staple food for Tibetans in Qinghai–Tibet, is adapted to a wide range of climatic conditions. This adaptation makes hull-less barley a unique resource for genetic research and crop improvement. Although genotypic differences in Cd responses and related molecular mechanism have been explored in barley [2,22,23,24], research specifically focusing on Cd tolerance in hull-less barley remains limited and warrants further investigation. Therefore, identifying suitable candidate genotypes through comprehensive physio-biochemical evaluations will effectively facilitate deeper molecular studies on Cd tolerance and accumulation.

In this study, we explore the physiological responses of 71 Tibetan hull-less barley genotypes under Cd stress to identify key traits and genotypes that are tolerant to Cd stress. Through a combination of morpho-physiological assessments, principal component analysis (PCA), and validation experiments, we assessed parameters including biomass production, photosynthesis parameters, elemental analysis, and antioxidant enzyme activities. Our results revealed significant variations in Cd tolerance among genotypes under Cd stress. These findings contribute to a deeper understanding of Cd stress responses and offer valuable insights for enhancing barley breeding programs, ultimately leading to the development of cultivars with improved resilience to Cd stress.

## 2. Results

### 2.1. Difference in Morpho-Physiological Response to Cd Stress Among Tibetan Hull-Less Barley Genotypes

**Figure 1:**
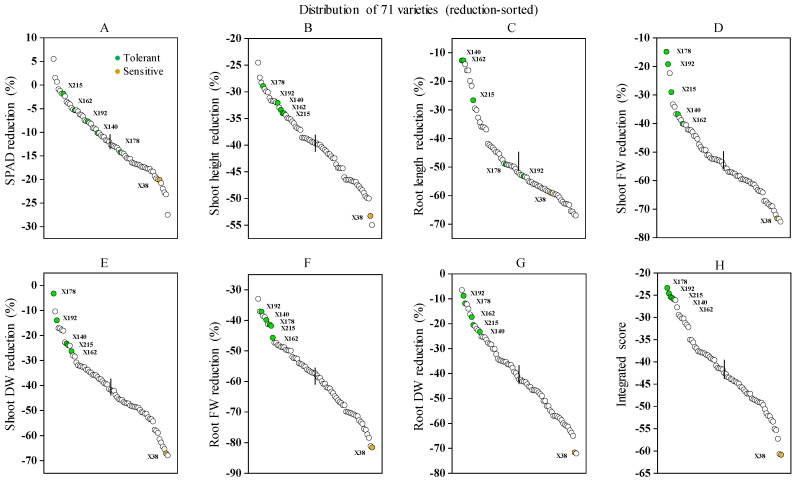
Differences in growth traits and integrated scores among 71 barley varieties under Cd stress. (**A**–**G**) Percentage reduction in various growth parameters after 15 days of exposure to 20 µM Cd stress compared to control conditions. (**H**) Integrated score based on these growth parameters; The growth parameters of barley seedlings were assessed as a percentage of the control to evaluate the impact of Cd stress. FW = fresh weight, DW = dry weight. ● Tolerant, ● sensitive, ○ not considered for further evaluation. Data are presented as means of three biological replicates (n = 3). The inset “|” indicates the least significant difference (LSD) at the 0.05 probability level between varieties.

As shown in Figure 1, Cd stress significantly inhibited the growth of seedlings across 71 barley genotypes. After 15 days of 20 μM Cd exposure, parameters such as SPAD value (chlorophyll meter readings), shoot height (SH), root length (RL), shoot fresh weight (SFW), root fresh weight (RFW), shoot dry weight (SDW), and root dry weight (RDW) decreased significantly by 11.74%, 39.69%, 48.09%, 52.88%, 58.39%, 40.59%, and 40.52%, respectively, compared to controls (Table 1). Cd toxicity symptoms varied significantly among genotypes, with X178, X192, X215, X140, and X162 being the least affected and X38 the most affected, particularly in biomass production and yellow necrotic patches. Reduction in growth parameters compared to controls was used to calculate the integrated score (IS, reduction % growth parameters compared to controls were used). A less negative IS indicates a smaller negative impact of Cd and greater tolerance, whereas a more negative IS reflects a stronger negative impact from Cd stress and greater sensitivity. From the preliminary selection experiment, 6 genotypes were chosen among the 71 genotypes, including 5 Cd-tolerant genotypes and 1 Cd-sensitive genotype according to the IS. The five tolerant genotypes—X178, X192, X215, X140, and X162—had IS values of −23.4, −24.6, −25.4, −25.6, and −25.9, respectively, and the one sensitive genotype—X38—had an IS of −60.9 (Figure 1H). The Shannon–Weaver diversity index for most parameters was ~2.04, while the SH and IS values were ~2.05 and 1.86 for RL, respectively (Table 1).

### 2.2. Identification of Cd-Tolerant and Sensitive Hull-Less Barley Genotypes

**Figure 2:**
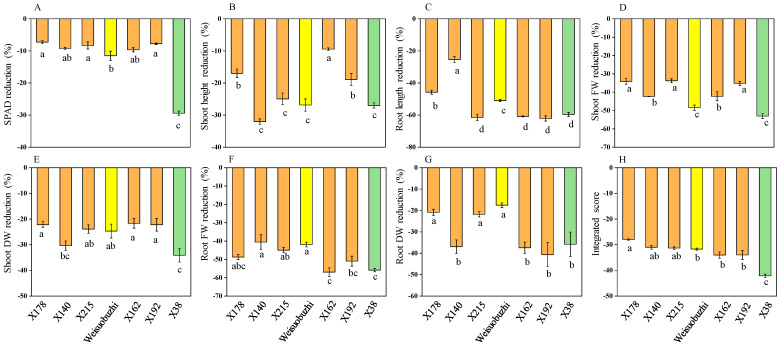
Differences in growth traits and integrated scores among the seven barley genotypes. (**A**–**G**) Percentage reduction in seven growth traits after 10 days of exposure to 20 µM Cd stress, expressed as a percentage of the control values. (**H**) Integrated scores for each genotype. FW = fresh weight; DW = dry weight. ■ Tolerant genotypes, ■ sensitive genotype, ■ check genotype (a Cd-tolerant reported previously [22]). Data are presented as means ± SD (n = 3). One-way ANOVA was used, and multiple comparisons were made using Duncan’s test. Different letters indicate significant differences at *p* < 0.05.

The effect of Cd toxicity on 6 selected genotypes, along with Weisuobuzhi (a Cd-tolerant check genotype), were assessed by evaluating the same morpho-physiological characteristics as preliminary selection after 10 days of 20 μM Cd exposure. Similar results were observed in the secondary selection, as shown in Figure 2 and Table 2. On average, SPAD value, SH, RL, SFW, RFW, SDW, and RDW were reduced by 11.9, 22.3, 53.3, 41.3, 48.6, 25.6, and 30.1%, respectively, compared with controls. Among the five tolerant genotypes, X178 exhibited a reduction in SPAD value, SFW, RFW, SDW, and RDW, with reductions of 7.26, 34.11, 48.74, 22.21, and 20.83%, respectively. In contrast, genotype X38 was the most sensitive to Cd toxicity, showing reductions in these parameters of 29.41, 53.07, 55.86, 34.14, and 35.77%, respectively, compared to controls. Compared with other tolerant genotypes, X178 had the highest IS, at −28.0, whereas sensitive X38 had the lowest IS, at −42.1. No visible leaf Cd toxicity indicators, such as necrotic patches, were observed in X178; however, X38 displayed visible necrotic patches under 20 μM Cd stress. 

### 2.3. Principal Component Analysis (PCA)

**Figure 3:**
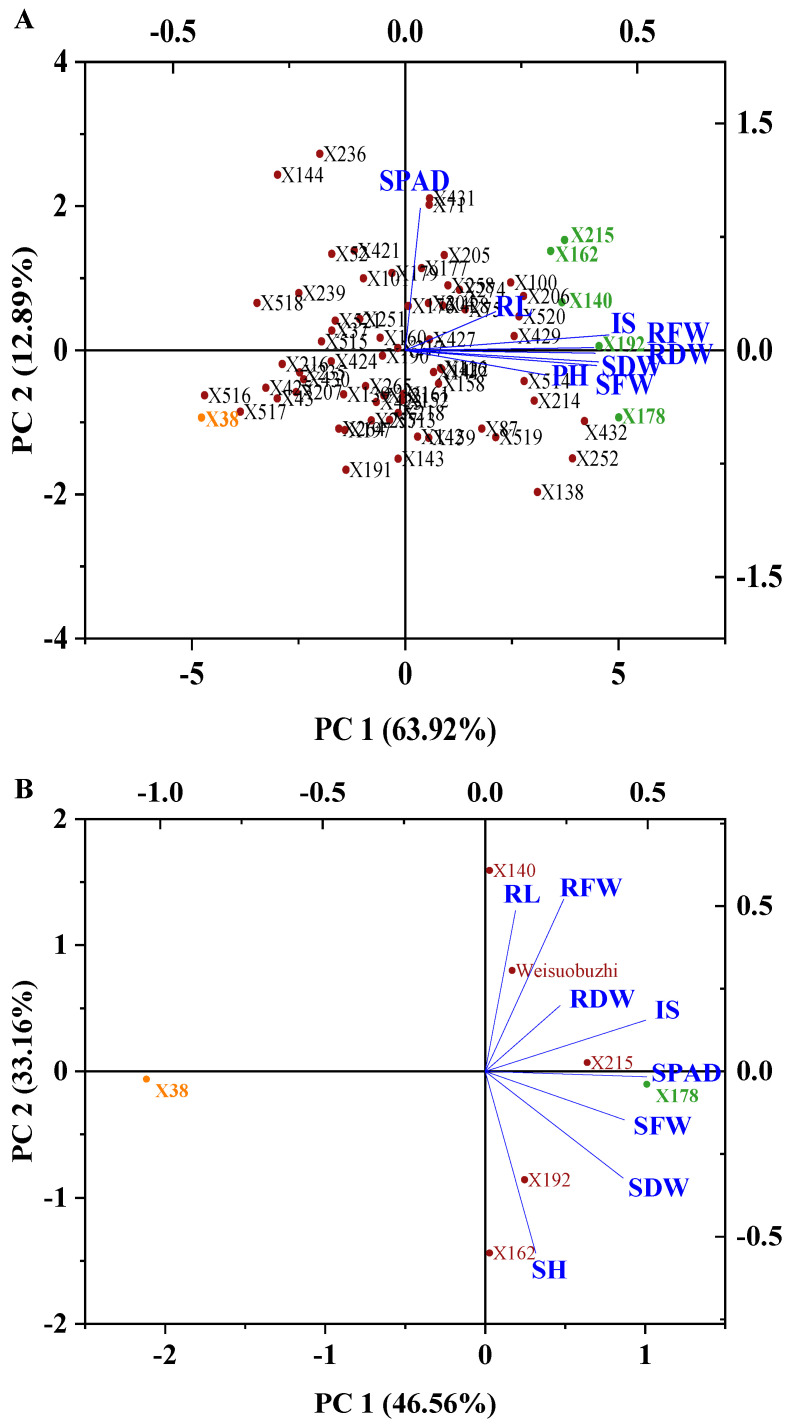
Bi-plot based on principle component analysis of reduction percentage of barley seedling morphological characters under 20 µM Cd stress conditions. (**A**) Preliminary selection (15 days after treatment), (**B**) secondary selection (10 days after treatment). (SPAD = SPAD value, SH = shoot height, RL = root length, SFW = shoot fresh weight, RFW = root fresh weight, RDW = root dry weight and SDW = shoot dry weight, IS = integrated score).

To investigate the specific contributions of the morpho-physiological traits, principal component analysis (PCA) was employed. The first principal component (PC1) explained 63.92% of the data variation, while the second principal component (PC2) explained 12.89%. Two PCs with eigenvalues above 1.0 together accounted for 76.81% variability (Table S1). The results showed that most characters contributed to PC1 under Cd stress. The bi-plots suggested that SPAD value, SH, RL, SFW, RFW, SDW, and RDW were major contributors to PC1. The analysis grouped the 71 barley genotypes based on Cd tolerance: Genotypes that were tolerant of Cd clustered to the right, genotypes that were neutral clustered in the middle, and Cd-sensitive genotypes clustered to the left (Figure 3A). PCA was performed on the relative values of morpho-physiological traits from a secondary selection experiment. PC1 explained approximately 46.56% of the variance, while PC2 accounted for 33.16% (Figure 3B). As in the preliminary selection, two PCs had eigenvalues above 1.0, explaining 79.72% of the variability (Table S2). The bi-plots indicated that the selected tolerant genotypes were positioned on the right side, while the sensitive genotype were on the left, consistent with the preliminary selection results. Therefore, X178 and X38, along with Weisuobuzhi, were selected for further validation experiments.

### 2.4. Comparative Analysis Between Cd-Tolerant X178 and -Sensitive X38 Genotypes

**Figure 4:**
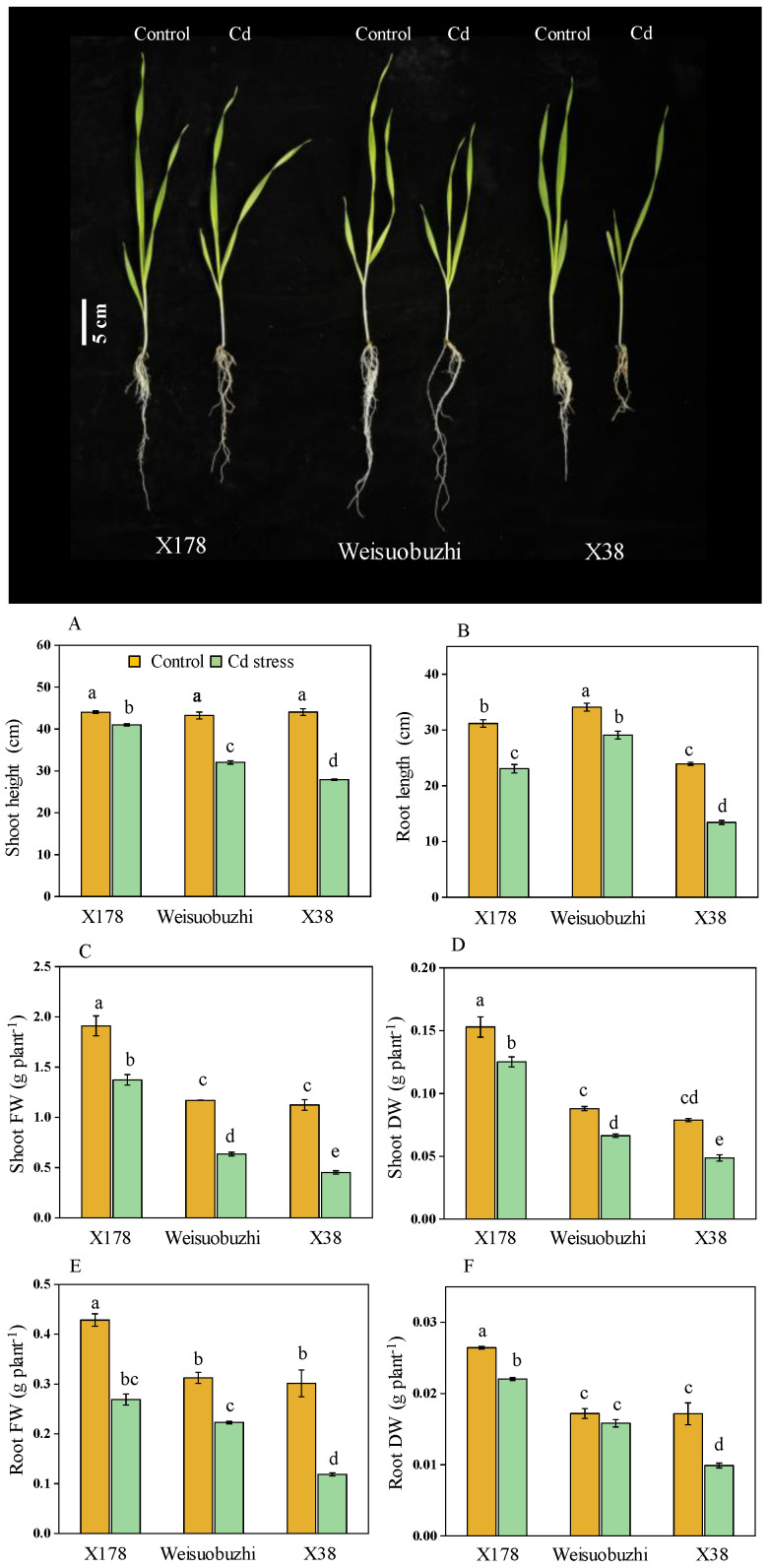
Phenotypical observation of X178, Weisuobuzhi, and X38 under control and 20 µM Cd stress (10 days after treatment, 15 days after germination). Differences in growth traits of tolerant genotype (X178), check genotype (Weisuobuzhi), and sensitive genotype (X38) varieties after 15 days under control and 20 µM Cd stress. (**A**–**F**) Six growth traits. FW = fresh weight, DW = dry weight. Data are presented as means ± SD (n = 3). One-way ANOVA was used, and multiple comparisons were made using Duncan’s test. Different letters indicate significant differences at *p* < 0.05.

In the preliminary and secondary selection experiment, we observed seedlings’ tolerance ability under Cd stress conditions, and finally, we selected Cd-tolerant X178 and -sensitive X38 for validation experiments. Under Cd toxicity (20 μM Cd exposure for 10 days), the X178 genotype exhibited less impact on most morphological parameters, showing relatively higher SH, SFW, SDW, and RDW compared to Weisuobuzhi, while the X38 genotype was most affected (Figure 4A–F). There were significant differences in SH, SFW, RFW, and SDW, but not RDW; the tolerant genotype X178 had reductions of 6.80%, 25.93%, 27.22%, 37.25%, 17.32%, and 16.66%, respectively, whereas the sensitive X38 had the highest reduction percentages of 36.49%, 43.82%, 59.25%, 59.56%, 38.33%, and 40.78%, respectively. The check genotype Weisuobuzhi had reductions of 25.80%, 14.68%, 45.65%, 8.47%, 24.44%, and 9.78%, respectively. Cd-tolerant X178 had the highest integrated score of −20.2; the sensitive X38 was the lowest, at −42.8. Furthermore, a two-way ANOVA was used to demonstrate the effects of genotype, treatment, and their interactions for physiological and biochemical measures in Table S3.

### 2.5. Genotype X178 Exhibits Better Photosynthetic Capacity Than X38

**Figure 5:**
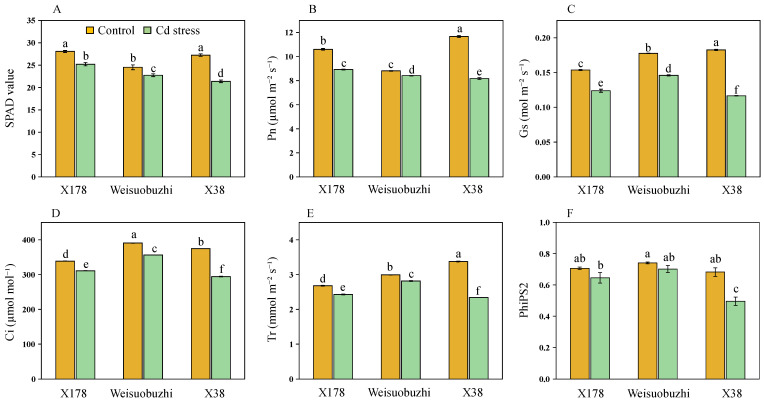
Effects of photosynthesis parameters of the tolerant genotype (X178), check genotype (Weisuobuzhi), and sensitive genotype (X38) under control and 20 µM Cd stress. (**A**) SPAD value; (**B**) net photosynthetic rate, Pn; (**C**) stomatal conductance, Gs; (**D**) intercellular carbon dioxide concentration, Ci; (**E**) transpiration rate, Tr; (**F**) effective photochemical efficiency of photosystem II, PhiPS2. Data are presented as means ± SD (n = 3). One-way ANOVA was used, and multiple comparisons were made using Duncan’s test. Different letters indicate significant differences at *p* < 0.05.

The physiological parameter-related data are shown in Figure 5A–F; all measured parameters showed reductions due to Cd stress, with the tolerant X178 generally exhibiting smaller reductions compared to the sensitive X38. Specifically, the values of all six parameters were significantly higher in X178 and lower in X38. Under Cd toxicity, leaf chlorophyll content (SPAD value) decreased by 10.22% in X178 and by 21.49% in X38 compared to their respective control. Photosynthetic and gas-exchange parameters, including net photosynthesis (Pn), stomatal conductance (Gs), intercellular carbon dioxide concentration (Ci), transpiration rate (Tr), and effective photochemical efficiency of photosystem II (PhiPS2), were decreased by 15.93, 13.14, 8.17, 7.74, and 8.75% in X178, respectively. In contrast, reductions in X38 constituted 32.97, 36.57, 21.65, 30.76, and 27.49%, respectively, under 20 μM Cd stress compared with their respective controls.

### 2.6. Genotype X178 Exhibits Better Nutrient Uptake than X38

**Figure 6:**
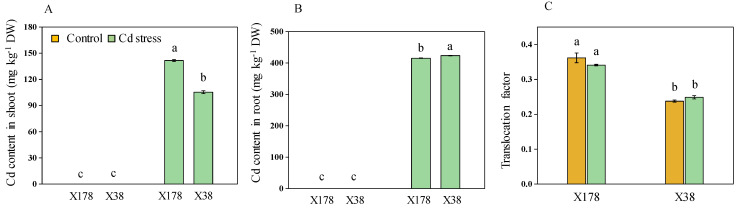
Cd content in shoot (**A**) and root (**B**) of barley seedlings after 15 days of 20 µM Cd treatment. DW, dry weight. Translocation factor = Cd concentration in shoot/Cd concentration in the root (**C**). Data are presented as means ± SD (n = 3). One-way ANOVA was used, and multiple comparisons were made using Duncan’s test. Different letters indicate significant differences at *p* < 0.05.

**Figure 7:**
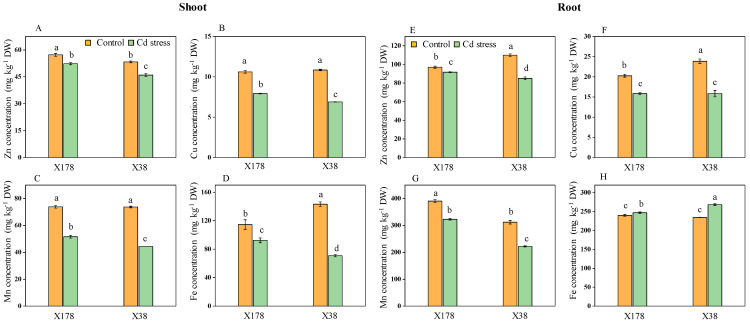
Effects of Cd stress on the concentrations of Zn, Cu, Mn, and Fe (mg kg^−1^ dry weight) in shoot (**A**–**D**) and root (**E**–**H**) of barley seedlings after 15 days of 20 µM Cd treatment. Data are presented as means ± SD (n = 3). One-way ANOVA was used, and multiple comparisons were made using Duncan’s test. Different letters indicate significant differences at *p* < 0.05.

The elemental concentrations in the shoots and roots of two genotypes, X178 and X38, were analyzed. Under control conditions, Cd concentration was very low in both genotypes, which might be seed-containing Cd. However, Cd levels increased significantly under Cd stress. For the Cd-tolerant X178 genotype, the Cd concentrations in the shoot and root were 141.63 mg kg^−1^ DW and 415.32 mg kg^−1^ DW, respectively, which were 25.64% higher in the shoot but 2.01% lower in the root compared to sensitive X38 (Figure 6). Specifically, X38 had Cd concentrations of 105.32 mg kg^−1^ DW in the shoot and 423.67 mg kg^−1^ DW in the root. The translocation factor was significantly higher in the X178 genotype, with a 27.1% increase in Cd translocation to the shoot compared to X38 under Cd stress. Significant variations were observed in the concentrations of microelements such as Zn, Cu, Mn, and Fe between the two barley genotypes. Both genotypes exhibited lower levels of these microelements in the shoots under Cd treatment compared to their controls, with X178 showing less reduction than X38 (Figure 7). Specifically, the concentrations of Zn, Cu, Mn, and Fe in the shoots of X178 were reduced by 8.59, 25.26, 30.12, and 19.06%, respectively, while X37 had reductions of 13.78, 36.44, 39.75, and 50.59%. In the roots, Zn, Cu, and Mn concentrations decreased by 5.51%, 21.68%, and 17.27% in X178, and by 22.69%, 33.69%, and 28.77% in X38. Notably, Fe concentrations in the roots increased by 3.01% in X178 and by 14.39% in X38. 

### 2.7. Genotype X178 Exhibits Better Oxidative Stress and Antioxidant Enzyme Activity Than X38

**Figure 8:**
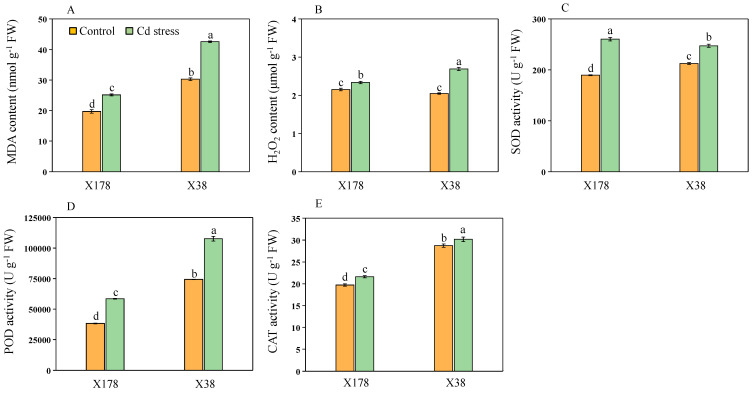
Effects of Cd on contents of malondialdehyde (MDA (**A**)), hydrogen peroxide (H_2_O_2_ (**B**)), and antioxidant enzyme activities of SOD (**C**), POD (**D**), and CAT (**E**) of leaves in barley seedlings after 10 days of Cd treatment. Data are presented as means ± SD (n = 3). One-way ANOVA was used, and multiple comparisons were made using Duncan’s test. Different letters indicate significant differences at *p* < 0.05.

Leaves from X178 and X38 were analyzed for oxidative stress induced by Cd treatment (Figure 8A,B). Under Cd treatment, malondialdehyde (MDA) content was significantly increased in both genotypes compared to their untreated controls. The sensitive genotype X38 exhibited a 40.42% increase in MDA content, while the tolerant X178 showed a 27.93% increase. Similarly, hydrogen peroxide (H_2_O_2_) content increased significantly in both genotypes. X38 displayed a 31.41% increase in H_2_O_2_ content, compared to 8.59% in X178. In response to stress, the activities of antioxidative enzymes superoxide dismutase (SOD), peroxidase (POD), and catalase (CAT) were significantly elevated in both genotypes compared to their controls (Figure 8C–E). The SOD activity in X178 increased by 37.33%, indicating a strong response to mitigating superoxide-induced oxidative stress, whereas X38 showed a 16.35% increase. The enhanced POD and CAT activities in X178 were 52.78% and 44.81%, respectively, while in X38, they were 9.84% and 5.04%.

## 3. Discussion

Plant Cd-tolerance and absorbance ability depend on genotypes, plant species physiological characteristics, morphological diversity [25,26,27], growth stage, and age of the plant [28]. This study observed that Cd stress significantly reduced chlorophyll content (SPAD value), SH, RL, SFW, RFW, SDW, and RDW in both selections (Table 1 and Table 2). Consistent with previous research, Cd exposure was found to impede early seedling growth and biomass accumulation compared to control, affecting shoot and root height, fresh weight, and dry weight [8,29,30,31]. Similar effects have been documented in barley [2,22,32]. This study employed a hydroponic culture system to assess Cd tolerance during the seedling stage, a method previously used to evaluate salt tolerance in wheat (*Triticum aestivum* L.) and Cd tolerance in barley [22,33,34]. Additionally, Cd tolerance in maize (*Zea mays* L.) has been evaluated through morphological characteristics, such as shoot and root length, and shoot and root fresh and dry weight [35]. Cd stress also resulted in a notable reduction in net photosynthesis (Pn), stomatal conductance (Gs), intercellular carbon dioxide concentration (Ci), transpiration rate (Tr), and effective photochemical efficiency of photosystem II (PhiPS2) values. Previous studies have shown that Cd significantly decreases photosynthetic parameters, with reductions observed in barley and rice (*Oryza sativa* L.) under Cd stress [36,37]. Metals like Cd affect plant gas-exchange characteristics by causing stomatal closure and reducing CO_2_ consumption [38,39].

PCA facilitates the grouping of observations by visually assessing similarities and differences using data sample plots [40]. The resulting bi-plot effectively grouped barley genotypes based on their response to Cd stress. While PCA alone may not fully capture genotype tolerance, its combination with IS enhances the detection of Cd tolerance in barley. Combining PCA with IS has proven useful in classifying Cd tolerance in maize, rice, and barley [41,42,43]. Previous research demonstrated that combining PCA with the temperature response index effectively categorized maize cold tolerance [41]. The accuracy of this study was documented by integrating results from the stress tolerance index with PCA [44]. Consequently, barley genotypes positioned on the right side of the bi-plot were classified as Cd-tolerant, whereas those on the left were deemed Cd-sensitive. Genotypes X178, X192, X215, X140, and X162 were initially identified as Cd-tolerant, while genotype X38 was categorized as Cd-sensitive based on IS ranking and PCA. Subsequent analysis confirmed X178 as tolerant and X38 as sensitive. Among 71 Tibetan hull-less barley genotypes, X178 and X38 consistently demonstrated tolerance and sensitivity, respectively. Further studies are needed to confirm these findings based on physiological and biochemical attributes.

Significant genotypic variations in Cd concentration were observed between X178 and X38 (Figure 6A,B). X178 exhibited the highest Cd concentration in seedling roots and the lowest in shoots compared to X38. Both genotypes effectively translocated Cd from roots to shoots, with the Cd-tolerant X178 transporting more Cd to the shoots than the Cd-sensitive X38 under Cd treatment (Figure 6C). Despite these differences in Cd translocation, X178 demonstrated Cd tolerance, as evidenced by better growth parameters and a lower integrated stress score compared to X38. It is likely that Cd-tolerant X178 exhibits fewer signs of Cd toxicity and may possess mechanisms that help it withstand Cd stress. The impact of Cd toxicity can vary depending on the genotype, exposure level, and plant species [45,46]. García de la Torre et al. [47] found that Cd content in roots and shoots does not always correlate with tolerance traits, suggesting that Cd accumulation alone may not reliably indicate Cd tolerance. Several transporters are involved in Cd uptake and translocation. Plasma membrane proteins such as OsHMA2, AtHMA2, and AtHMA4 facilitate the loading of Cd^2+^ into the xylem, playing a critical role in controlling Cd translocation from roots to shoots [48,49]. Additionally, Cd tolerance is regulated by a complex network of transporters. For example, AtHMA3 and OsHMA3 are located in the tonoplast and contribute to Cd sequestration in vacuoles [50,51]. Various members of the ABC transporter family, such as AtABCC1 and AtABCC2, have also been implicated in protective roles during similar processes [52].

Cd is initially absorbed by plant roots, often leading to higher accumulation in roots compared to above-ground parts [53]. Transporters for essential elements like Fe and Zn may also facilitate Cd transport across root cell membranes [54,55,56]. Significant differences in the concentrations of microelements between the two barley genotypes were observed compared to their controls (Figure 7). Cd can impair the uptake, use, and storage of mineral nutrients in plants such as Ca, Mg, Cu, Zn, Mn, K, P, S, N, Si, and Fe [57,58,59,60]. For instance, tomato plants exposed to 100 µM Cd showed lower Zn, Cu, and Mn content but increased Fe content [45]. Due to their poor selectivity for divalent metal cations (Fe^2+^, Mn^2+^, Cd^2+^, and Zn^2+^), NRAMP and ZIP family transporters are primarily responsible for Cd^2+^ absorption. For example, HvNRAMP5 in barley and OsZIP3 in rice have been shown to be involved in the uptake and transport of Cd^2+^ [19,61]. Further molecular research is needed to better understand the mechanisms of nutrient uptake and transport in response to Cd stress in Cd-tolerant genotypes.

**Figure 9:**
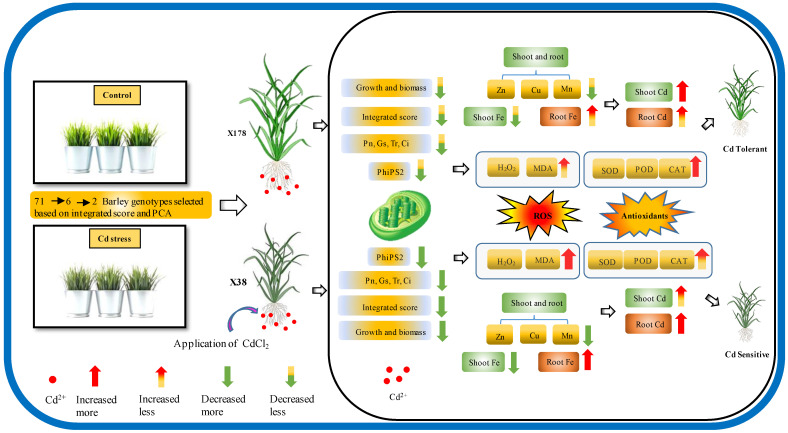
Effects of Cd toxicity on morpho-physiological, elemental (shoot and root), oxidative, and antioxidant (leaves) parameters of barley. Net photosynthetic rate (Pn), stomatal conductance (Gs), intercellular carbon dioxide concentration (Ci), transpiration rate (Tr), effective photochemical efficiency of photosystem II (PhiPS2), malondialdehyde (MDA), hydrogen peroxide (H_2_O_2_), superoxide dismutase (SOD), peroxidase (POD), and catalase (CAT). Each parameter changes in measured parameters under Cd treatment compared to the control.

Plant growth and development are significantly affected by oxidative stress caused by Cd toxicity [14]. Previous studies have shown that Cd exposure leads to the overproduction of ROS [13,62]. Our study found that Cd stress resulted in oxidative stress in barley leaves, reflected by increased MDA and H_2_O_2_ contents in plants, with higher levels in sensitive genotype X38 than tolerant genotype X178 (Figure 8A,B). Similar findings were reported in other studies involving Cd-tolerant and sensitive barley genotypes [63,64]. Plants established both the enzymatic and non-enzymatic self-protective mechanisms to scavenge excess ROS and reduce oxidative stress [62]. In this study, activities of antioxidant enzymes such as SOD, POD, and CAT were enhanced in both genotypes under Cd stress, especially in the Cd-tolerant genotype X178 (Figure 8C–E). Recent studies have provided further insights into the molecular regulation of these antioxidant enzymes under Cd stress. For example, Cd exposure has been shown to upregulate several stress-responsive genes, including those encoding SOD and CAT, which play a key role in counteracting ROS-induced damage [65,66]. Additionally, research has identified the involvement of transcription factors, such as MYB and WRKY families, in regulating antioxidant gene expression in response to heavy metal stress [67,68,69]. Notably, the WRKY transcription factor has been shown to promote antioxidant enzyme expression under Cd stress in poplar, thereby enhancing tolerance to oxidative damage [70]. Moreover, X178 exhibited less biomass reduction, higher antioxidant enzyme activities, and reduced oxidative stress compared to the untreated control, outperforming X38 (Figure 9). In contrast, the Cd-sensitive genotype showed decreased antioxidant responses and increased oxidative damage. The differential response observed between X38 and X178 suggests that Cd tolerance may be linked to the fine-tuning of antioxidant enzyme activities. These findings are consistent with previous reports indicating that tolerant barley genotypes maintain higher enzyme activity and lower levels of lipid peroxidation, reflecting a more robust antioxidant response, while sensitive genotypes exhibit increased oxidative damage and reduced enzyme production [71,72]. Future research should focus on elucidating the genetic and molecular mechanisms underlying antioxidant enzyme regulation in response to Cd, particularly the roles of key transcription factors and signaling pathways.

## 4. Materials and Methods

### 4.1. Experimental Design and Cultural Condition

Hydroponic experiments were conducted during 2023–2024 on the Zijingang Campus, Zhejiang University, China. The experiment design employed a split-plot approach, with treatments as the main plot and genotypes as the sub-plot, including three replications with five plants per replication. Uniform six-day-old barley seedlings of each genotype were selected and transplanted into 20 L containers in a 19.5 L basic nutrient solution (BNS). The BNS contained (mg L^−1^): (NH_4_)_2_SO_4_ 48.2, MgSO_4_ 65.9, K_2_SO_4_ 15.9, KNO_3_ 18.5, Ca(NO_3_)_2_ 59.9, KH_2_PO_4_ 24.8, Fe-citrate 5, MnCl_2_·4H_2_O 0.9, ZnSO_4_ 7H_2_O 0.11, CuSO_4_·5H_2_O 0.04, HBO_3_ 2.9, H_2_MoO_4_ 0.01 [2]. The pH of the solution was adjusted to 5.8 ± 0.1 with NaOH or HCl as necessary. The solution was continuously aerated and was renewed every 5 days. A total of 71 hull-less barley genotypes were used in the preliminary experiment. Each genotype’s seeds were sterilized in 2% (*v*/*v*) hydrogen peroxide (H_2_O_2_) and then washed carefully with distilled water. Sterilized seeds were germinated on sterilized filter paper in a Petri dish in a plant growth chamber (22/18 °C day/night) in the dark for three days and protected for the next four days in light environments. Cd as CdCl_2_ was used to prepare a stock solution of 50 mM and added 15 days after transplanting to individual containers of the basic nutrient solution as two levels of treatments—0 (control, without Cd) and 20 μM Cd. In the second selection trial, five genotypes—X178, X192, X215, X140, and X162—were selected as Cd-tolerant and X38 as Cd-sensitive. Therefore, these genotypes were selected for additional evaluation of Cd-tolerance, along with Weisuobuzhi (Cd-tolerant) as a check genotype [22]. Seeds of each genotype were sterilized and germinated as described previously. On the 5th day after transplanting, Cd as CdCl_2_ was added as described above. The validation experiment used tolerant X178 and sensitive X38 genotypes along with Weisuobuzhi from the secondary selection experiment. All the experiment conditions were the same as described for the previous selection experiment.

### 4.2. Growth Measurement

Plants were harvested 15 days after treatments and thoroughly washed with ddH_2_O to remove external contaminants. After drying the samples with tissue paper, shoot height (SH) and root length (RL) were determined. Seedling samples were then separated into roots and shoots, and both root fresh weight (RFW) and shoot fresh weight (SFW) were recorded. The roots and shoots of each genotype were dried in a hot-air oven at 65 °C for 3 consecutive days until a constant weight was reached, after which root dry weight (RDW) and shoot dry weight (SDW) were measured. These data and relative values were used to calculate the integrated score (IS), which, based on Chen et al. [22] with some modifications, was determined using the following formula: IS = [(SPAD values × 1/7) + (SH × 1/7) + (RL × 1/7) + (SFW × 1/7) + (RFW × 1/7) + (SDW × 1/7) + (RDW × 1/7)]. Relative values for each parameter were calculated using relative values = [{(Cd − CK)/CK} × 100], where CK and Cd represent the control and Cd stress conditions, respectively. The Shannon–Weaver diversity index (H′) was calculated using: H′ = −SUM (pi × lnpi), where pi is the relative abundance of individual group of accessions tested, and lnpi is the natural logarithm of that proportion value [73]. In the second experiment, plants were harvested 10 days after Cd treatment, and the same growth parameters were measured as described in the preliminary selection experiment. These data were utilized to select Cd-tolerant barley genotypes for validation. In the validation experiment, after 15 days of Cd treatment, plants were harvested, and all parameters, including SH, RL, RFW, SFW, RDW, and SDW, were measured according to the previously described methods.

### 4.3. Photosynthetic and Gas-Exchange Feature Measurement

To measure SPAD values (chlorophyll meter readings) of the fully extended leaves (the first from the apex), a SPAD-502 chlorophyll meter (Minolta Corporation, Ltd., Osaka, Japan) was used. The LI-6400 portable photosynthesis system (LI-COR Biosciences, Lincoln, NE, USA) was used to measure the net photosynthetic rate (Pn), stomatal conductance (Gs), transpiration rate (Tn), and intracellular CO_2_ concentration (Ci). The effective photochemical efficiency of photosystem II (PhiPS2) was determined using an LI-600 porometer/fluorometer (LI-COR Biosciences, Lincoln, NE, USA). Data were recorded from the 2nd fully expanded leaves. All the measurements were taken 10 days after Cd treatment.

### 4.4. Element Concentration Measurement

To determine the elemental concentrations, the seedlings were collected 15 days after Cd treatment and separated into roots and shoots, then root samples were immersed in 20 mM Na_2_EDTA for 3 h and washed away with deionized water to eliminate the ions attached to the root’s surface. The samples were dried at 65 °C for 72 h to constant weight before further analysis. The dried root and shoot samples were ground, weighted, and completely digested in 2 mL of 70% nitric acid (HNO_3_) at 120 °C on an aluminum block heater (Dry ThermoUnit DTU-2CN), then diluted with deionized water. Concentrations of ions such as Cd, Zn, Cu, Mn, and Fe were determined using inductively coupled plasma–mass spectrometry ICP-MS (ICAP RQ, Thermo Fisher, Waltham, MA, USA).

### 4.5. Oxidative Stress and Antioxidant Enzyme Measurement

Fresh fully expended upper second leaves were sampled 10 days after Cd treatment, instantly placed into liquid nitrogen, and stored at −80 °C. The lipid peroxidation capacity measurement was done following the technique outlined by Ahmed et al. [74] and Dong et al. [75]. An indicator of lipid peroxidation and malondialdehyde (MDA) levels were measured by a Synergy H1 microplate reader (BioTek, Shoreline, WA, USA) at a wavelength of 532 nm. This measurement was performed with a destruction coefficient of 155 mM^−1^ cm^−1^. H_2_O_2_ extraction and determination were performed with the procedure outlined in Ahmed et al. [76]. Approximately 0.1 g of fresh leaf sample were homogenized with 5 mL of 50 mM sodium phosphate buffer (PBS, pH 7.8) and 0.5 mM of ethylenediaminetetraacetic acid (EDTA) by a mortar and pestle. After the homogenate was centrifuged at 12,000× *g* for 30 min at 4 °C, the antioxidant enzyme activities were measured using the obtained supernatant. Antioxidant enzyme activities of superoxide dismutase (SOD), peroxidase (POD), and catalase (CAT) were determined by the procedures employed by Ahmed et al. [77] and Ibrahim et al. [78].

### 4.6. Statistical Analysis

All collected data presented are the mean values of three replicates. MS Excel was used for the processing and analysis of experimental data. One-way analysis of variance (ANOVA) was conducted and multiple comparisons by Duncan’s post hoc test were used to estimate the significance of the difference. All statistical analysis was executed by IBM SPSS version 26.0 software. For plotting the results, Origin 2021 (OriginLab, Northampton, MA, USA) was used.

## 5. Conclusions

This investigation elucidates significant variations in the growth and development of barley seedlings, particularly in biomass characteristics and physiological responses to Cd stress among the Tibetan hull-less barley genotypes concerning molecular mechanisms of Cd tolerance. Recently, there has been growing interest in the molecular mechanisms underlying Cd tolerance, and we have identified a promising candidate that could serve as a foundation for future molecular investigations. This finding provides critical insights into the mechanisms of Cd tolerance. The validation experiments strengthened the preliminary and secondary findings, highlighting that genotypes X178 exhibit remarkable tolerance, characterized by better biomass production, higher chlorophyll content, and more efficient antioxidant defense mechanisms compared to sensitive genotypes. This study represents an initial screening of Cd tolerance and lays the foundation for future investigations into the molecular mechanisms involved, including the roles of specific genes, proteins, and signaling pathways that govern Cd uptake, detoxification, and compartmentalization within plant tissues. Ultimately, this research contributes to the development of barley varieties that can be cultivated in Cd-contaminated soils.

## Figures and Tables

**Table 1:** Effect of Cd on growth traits and integrated score of barley seedlings in the preliminary selection experiment.

Reduction Percentage	SPAD Value	Shoot Height	Root Length	Shoot Fresh Weight	Root Fresh Weight	Shoot Dry Weight	Root Dry Weight	Integrated Score
Mean	−11.74	−39.69	−48.09	−52.88	−58.39	−40.59	−40.52	−41.7
Min	5.54	−24.53	−12.71	−14.84	−32.96	−3.23	−6.50	−23.4
Max	−27.47	−54.97	−66.97	−74.38	−81.56	−67.87	−72.07	−60.9
Diversity index	2.04	2.07	1.86	2.02	2.03	2.03	2.04	2.05
CV %	57.60	17.07	29.55	24.36	20.26	34.64	39.17	21.5
Between genotypes	**	**	**	**	**	**	**	**
Between treatments	**	**	**	**	**	**	**	**

**, Significance at the 0.01 probability level between genotypes and treatment. Data represented as the percentage of control (%). For each genotype, three biological replicates were used.

**Table 2:** Effect of Cd on growth traits and integrated score of barley seedlings in the secondary selection experiment.

Reduction Percentage	SPAD Value	Shoot Height	Root Length	Shoot Fresh Weight	Root Fresh Weight	Shoot Dry Weight	Root Dry Weight	Integrated Score
Mean	−11.9	−22.3	−52.3	−41.3	−48.6	−25.6	−30.1	−33.2
Min	−7.3	−9.4	−25.4	−33.8	−40.6	−21.7	−17.5	−28.0
Max	−29.4	−32.0	−62.1	−53.1	−56.9	−34.1	−40.6	−42.1
CV (%)	66.2	34.2	25.6	18.1	13.3	18.7	32.0	13.4
Between genotypes	**	**	**	**	**	**	**	**
Between treatments	**	**	**	**	**	*	**	**

**, *, Significance at the 0.01 and 0.05 probability level between genotypes and treatments. Data represented as the percentage of control (%). For each genotype, three biological replicates were used.

## Data Availability

The original contributions presented in the study are included in the article; further inquiries can be directed to the corresponding author.

## Supplementary Materials

The following supporting information can be downloaded at: https://www.mdpi.com/article/10.3390/plants13243593/s1, Table S1. Principal component analysis of 8 morphological characters of 71 barley genotypes under Cd stress. Table S2. Principal component analysis of 8 morphological characters of 7 barley genotypes under Cd stress. Table S3. Effects of genotype, treatment, and their interactions for the parameters considered by two-way analysis of variance (ANOVA). G × T represents the interaction between genotype and treatment; numbers represent F values at 0.05 probability level; ** represent significance < 0.01.
