# Supplementary material for: Comprehensive Physio-Biochemical Evaluation Reveals Promising Genotypes and Mechanisms for Cadmium Tolerance in Tibetan Hull-Less Barley
Source: Plants (Basel). 2024 Dec 23;13(24):3593. doi: 10.3390/plants13243593 (PMC11676794; doi:10.3390/plants13243593)
Supplement: Supplementary file 1 [file plants-13-03593-s001.zip › plants-3393618-supplementary.pdf]

Supplementary Information (SI)

Table S1. Principal component analysis of 8 morphological characters of 71 barley genotypes under Cd stress

PC	Eigenvalue	Percentage		Characters	Coefficients	
		Variance	Cumulative		PC1	PC2
1	5.113	63.92%	63.92%	SPAD	0.033	0.939
2	1.031	12.89%	76.81%	SH	0.304	-0.164
3	0.864	10.80%	87.61%	RL	0.195	0.255
4	0.612	7.64%	95.25%	SFW	0.413	-0.101
5	0.284	3.56%	98.81%	SDW	0.416	-0.077
6	0.063	0.78%	99.59%	RFW	0.406	0.018
7	0.033	0.41%	100.00%	RDW	0.409	-0.020
8	0.000	0.00%	100.00%	IS	0.439	0.101

SPAD = SPAD value, SH = shoot height, RL = root length, SFW = shoot fresh weight, RFW = root fresh weight, RDW = root dry weight and SDW = shoot dry weight, IS= integrated score

Table S2. Principal component analysis of 8 morphological characters of 7 barley genotypes under Cd stress

PC	Eigenvalue	Percentage		Characters	Coefficients	
		Variance	Cumulative		PC1	PC2
1	3.725	46.56%	46.56%	SPAD	0.498	-0.017
2	2.653	33.16%	79.72%	SH	0.156	-0.549
3	0.970	12.13%	91.85%	RL	0.093	0.487
4	0.422	5.28%	97.12%	SFW	0.428	-0.147
5	0.224	2.80%	99.92%	SDW	0.242	0.521
6	0.006	0.08%	100.00%	RFW	0.424	-0.323
7	0.000	0.00%	100.00%	RDW	0.231	0.199
8	0.000	0.00%	100.00%	IS	0.494	0.155

SPAD = SPAD value, SH = shoot height, RL = root length, SFW = shoot fresh weight, RFW = root fresh weight, RDW = root dry weight and SDW = shoot dry weight, IS= integrated score

Table S3. Effects of genotype, treatment, and their interactions for the parameters considered by two-way analysis of variance (ANOVA). G × T represents the interaction between genotype and treatment; numbers represent F values at 0.05 probability level; ** represent significance < 0.01

Parameters	Shoot			Root		
	**Genotype**	**Treatment**	**G × T**	**Genotype**	**Treatment**	**G × T**
SPAD	46.667**	163.438**	19.286**	-	-	-
S/R L	150.030**	322.207**	151.569**	177.679	216.075**	3.598**
S/R FW	142.359	71.237**	0.862**	56.117	85.377**	0.391**
S/R DW	190.408	28.139**	0.048**	134.797	40.055**	2.390**
Pn	121.900**	1158.020**	142.497**	-	-	-
Gs	0.487**	131.238**	30.549	-	-	-
Ci	238.002**	7737.537**	1864.944**	-	-	-
Tr	882.901**	4335.365**	1925.341**	-	-	-
PhiPS2	8.516	17.196**	4.390**	-	-	-
Cd content	229.786**	10623.551**	229.738**	33.858**	340432.474**	33.596**
Zn content	44.381	62.987**	2.449**	5.366**	128.713**	53.631**
Cu content	13.797**	954.554**	35.048**	8.851**	104.753**	8.808**
Mn content	17.621**	861.432**	16.234**	281.759	217.947**	4.423**
Fe content	0.529**	99.046**	28.015	14.108**	92.813**	39.016**
MDA content	878.021**	349.397**	51.877**	-	-	-
H_2O_2 content	10.696**	118.743**	36.448**	-	-	-
SOD activity	2.934**	323.156**	37.601	-	-	-
POD activity	1431.476**	563.883**	33.908**	-	-	-
CAT activity	425.555	15.689**	0.300**	-	-	-

Note: SPAD value, SPAD; shoot/root length, S/R L; shoot/root fresh weight, S/R FW, and shoot/root dry weight, S/R DW; net photosynthetic rate, Pn; stomatal conductance, Gs; intercellular carbon dioxide concentration, Ci; Transpiration rate, Tr; effective photochemical efficiency of photosystem II, PhiPS2; malondialdehyde, MDA; hydrogen peroxide, H_2O_2; superoxide dismutase, SOD; peroxidase, POD, and catalase, CAT.
